# Expression of the RNA-binding protein RBM3 is associated with a favourable prognosis and cisplatin sensitivity in epithelial ovarian cancer

**DOI:** 10.1186/1479-5876-8-78

**Published:** 2010-08-20

**Authors:** Åsa Ehlén, Donal J Brennan, Björn Nodin, Darran P O'Connor, Jakob Eberhard, Maria Alvarado-Kristensson, Ian B Jeffrey, Jonas Manjer, Jenny Brändstedt, Mathias Uhlén, Fredrik Pontén, Karin Jirström

**Affiliations:** 1 Center for Molecular Pathology, Department of Laboratory Medicine, Lund University, Skåne University Hospital, Malmö, Sweden; 2 UCD School of Biomolecular and Biomedical Science, UCD Conway Institute, University College Dublin, Belfield, Dublin 4, Ireland; 3 Division of Oncology, Department of Clinical Sciences, Lund University, Skåne University Hospital, Lund, Sweden; 4 School of Medicine and Medical Science, Conway Institute, University College Dublin, Dublin, Ireland; 5 Division of Surgery, Department of Clinical Sciences, Lund University, Skåne University Hospital, Malmö, Sweden; 6 The Malmö Diet and Cancer Study, Skåne University Hospital, Malmö, Sweden; 7 Department of Biotechnology, AlbaNova University Center, Royal Institute of Technology, Stockholm, Sweden; 8 Department of Genetics and Pathology, Rudbeck Laboratory, Uppsala University, Uppsala, Sweden

## Abstract

**Background:**

We recently demonstrated that increased expression of the RNA-binding protein RBM3 is associated with a favourable prognosis in breast cancer. The aim of this study was to examine the prognostic value of RBM3 mRNA and protein expression in epithelial ovarian cancer (EOC) and the cisplatin response upon RBM3 depletion in a cisplatin-sensitive ovarian cancer cell line.

**Methods:**

RBM3 mRNA expression was analysed in tumors from a cohort of 267 EOC cases (Cohort I) and RBM3 protein expression was analysed using immunohistochemistry (IHC) in an independent cohort of 154 prospectively collected EOC cases (Cohort II). Kaplan Meier analysis and Cox proportional hazards modelling were applied to assess the relationship between RBM3 and recurrence free survival (RFS) and overall survival (OS). Immunoblotting and IHC were used to examine the expression of RBM3 in a cisplatin-resistant ovarian cancer cell line A2780-Cp70 and its cisplatin-responsive parental cell line A2780. The impact of RBM3 on cisplatin response in EOC was assessed using siRNA-mediated silencing of RBM3 in A2780 cells followed by cell viability assay and cell cycle analysis.

**Results:**

Increased RBM3 mRNA expression was associated with a prolonged RFS (HR = 0.64, 95% CI = 0.47-0.86, *p = 0.003*) and OS (HR = 0.64, 95% CI = 0.44-0.95, *p = 0.024*) in Cohort I. Multivariate analysis confirmed that RBM3 mRNA expression was an independent predictor of a prolonged RFS, (HR = 0.61, 95% CI = 0.44-0.84, *p = 0.003*) and OS (HR = 0.62, 95% CI = 0.41-0.95; *p = 0.028*) in Cohort I. In Cohort II, RBM3 protein expression was associated with a prolonged OS (HR = 0.53, 95% CI = 0.35-0.79, *p = 0.002*) confirmed by multivariate analysis (HR = 0.61, 95% CI = 0.40-0.92, *p = 0.017*). RBM3 mRNA and protein expression levels were significantly higher in the cisplatin sensitive A2780 cell line compared to the cisplatin resistant A2780-Cp70 derivative. siRNA-mediated silencing of RBM3 expression in the A2780 cells resulted in a decreased sensitivity to cisplatin as demonstrated by increased cell viability and reduced proportion of cells arrested in the G2/M-phase.

**Conclusions:**

These data demonstrate that RBM3 expression is associated with cisplatin sensitivity *in vitro *and with a good prognosis in EOC. Taken together these findings suggest that RBM3 may be a useful prognostic and treatment predictive marker in EOC.

## Background

Epithelial ovarian cancer (EOC) is the leading cause of death from gynaecological malignancy and the fifth most common cause of cancer-related death in women. The poor ratio of survival to incidence in EOC is related to the high percentage of cases diagnosed at an advanced stage and the lack of effective therapies for advanced refractory disease. Despite improvements in surgical techniques and the advent of more targeted therapeutic agents, five year survival rates for EOC are only 45% [1]. Such poor statistics indicate an urgent requirement to improve on current understanding of the molecular mechanisms underlying EOC, so as to develop better early diagnostic and prognostic biomarkers. In addition, accurate predictive biomarkers are required to guide current treatment protocols, as well as to guide the development and application of new targeted therapies.

Since its inception over 40 years ago, the platinum-based agent cisplatin has had a major impact on cancer therapy, particularly in the treatment of testicular and ovarian cancer [2]. Standard treatment for advanced EOC involves surgical debulking followed by adjuvant chemotherapy with a combination of a platinum compound (cisplatin or carboplatin) and taxane [3]. Despite an initial response to cisplatin treatment, many patients with EOC develop resistance to the drug and relapse within a few years [4]. Cisplatin acts by forming covalent bonds with purine DNA bases which causes cross-linking of DNA and results in activation of several signal transduction pathways involved in DNA-damage repair, cell cycle arrest and apoptosis [2,5,6]. Several mechanisms have been implicated in cisplatin resistance, i.e. decreased drug uptake, insufficient DNA-binding of the drug, increased DNA-repair of cisplatin adducts and failure of induction of apoptosis, reviewed in [2,5,7].

The RNA binding motif protein 3, RBM3, is a glycine rich protein containing a RNA-recognition motif (RRM) through which it binds to both to DNA and RNA [8]. Proteins containing specific RRMs play an important role in the stabilization of mRNA by reversibly binding to conserved sequence elements, most often AU-rich elements (AREs), in the untranslated regions (UTRs) of the mRNA resulting in either stabilization or destabilization of the mRNA [9]. The RBM proteins, 10 of which have been described, contain between one and four copies of the RRM consensus sequence [10]. The RRM domain is evolutionary conserved across species and found in virtually every cellular organelle in which RNA is present suggesting an important but as yet not fully understood functionality [10]. RBM3, initially identified in a fetal brain cDNA library [11] is one of three X-chromosome related RBM-genes (RBMX, RBM3, RBM10) mapped to Xp11.23 [12] and is expressed in various human fetal tissues as well as being one of the earliest proteins induced by hypothermia [13]. Following an antibody-based proteomics biomarker discovery strategy using the Human Protein Atlas (HPA) (http://www.proteinatlas.org) [14,15] we recently demonstrated an association between nuclear RBM3 expression in breast cancer and a significantly improved survival, particularly in estrogen receptor (ER) positive tumors [16].

In the present study, the prognostic value of RBM3 was examined in two independent EOC cohorts, both at the mRNA levels (Cohort I) and protein levels (Cohort II), whereby RBM3 was found to be associated with a good prognosis in both cohorts. RBM3 expression was also examined *in vitro *using the cisplatin sensitive ovarian cancer cell line A2780 and its cisplatin resistant derivative A2780-Cp70. The relationship between RBM3 expression and cisplatin response *in vitro *was examined using small interfering RNA (siRNA) mediated RBM3 knockdown in the A2780 cells which resulted in a decreased sensitivity to cisplatin as demonstrated by an increased cell viability and reduced proportion of cells G2/M-phase arrest following cisplatin treatment.

## Methods

### Patients

#### Cohort I

Cohort I comprised of 285 cases of serous and endometroid carcinoma of the ovary, fallopian tube and peritoneum. The cohort has been described previously [17]. The majority of patients underwent laparotomy for staging and debulking and subsequently received first-line platinum/taxane based chemotherapy. In most cases, tumor tissue was excised at the time of primary surgery, prior to the administration of chemotherapy. Eighteen patients who had received neoadjuvant platinum based chemotherapy were also included in the cohort but excluded from this study hence the total number or patients examined was 267. Optimal debulking was defined as less than 1 cm (diameter) residual disease, and sub-optimal debulking was more than 1 cm (diameter) residual disease. Recurrence-free survival (RFS) was defined as the time interval between the date of diagnosis and the first confirmed sign of disease recurrence based on GCIG definitions. Overall survival (OS) was defined as the time interval between the date of histological diagnosis and the date of death from any cause. Median follow up was 29 months (range 0-214 months).

RNA was extracted from tumors and hybridized to Affymetrix U133 Plus 2 arrays as previously described [17]. Complete expression data were downloaded from GEO (http://www.ncbi.nlm.nih.gov/geo) (accession GSE9899). R package ''Affy'' (http://www.bioconductor.org) was used to normalize the CEL files using the RMA method [18]. For RBM3 analysis normalized gene expression values were extracted from the dataset and used without modification. Tumor samples were classified using a previously published method [19].

#### Cohort II

This cohort is a merge of all incident cases of epithelial ovarian cancers in the large, population-based prospective cohort studies Malmö Diet and Cancer Study [20] (n = 101) and Malmö Preventive Medicine Study [21] (n = 108) until Dec 31^st ^2008. Thirty-five patients participated in both studies, and archival tumor tissue could be retrieved from 154 of the total number of 174 cases. After a median follow-up of 2.65 years (range 0-21), 105 patients (68.2%) were dead and 49 (31.8%) alive.

All tumors were re-evaluated regarding histological subtype and histological grade. Information regarding clinical stage was obtained from the medical charts, following the standardized FIGO classification of tumor staging. Information on residual tumor after surgery was not available. Standard adjuvant therapy was platinum-based chemotherapy, from the 1990s given in combination with paclitaxel.

### Tissue microarray construction

Prior to TMA-construction, all cases were histopathologically re-evaluated on haematoxylin and eosin stained slides. Areas representative of cancer were then marked and TMAs constructed as previously described [22]. In brief, 2-4 1.0 mm cores were taken from each tumor and mounted in a new recipient block using a semi-automated arraying device (TMArrayer; Pathology Devices, Inc, Westminster, MD, USA).

### RBM3 antibody generation and immunohistochemistry

PrEST [23,24] antigen was injected subcutaneously into BALB/c mice (4-6 weeks old, female) at three weeks intervals. The antigen was mixed with complete Freund's adjuvant for the first injection and incomplete Freund's adjuvant for the following injections. Three days before infusion, the mouse was last challenged with antigen intravenously. Hybridomas were generated by fusion of mouse splenocytes with the Sp2/0 myeloma cell line. Cell lines that showed positive results in ELISA, Western blot (WB) and immunohistochemistry (IHC) were selected for subcloning.

For immunohistochemical analysis of RBM3 in Cohort II, 4 μm TMA-sections were automatically pretreated using the PT-link system (DAKO, Copenhagen, Denmark) and then stained in a Techmate 500 (DAKO, Copenhagen, Denmark) with the mouse monoclonal anti-RBM3 antibody (AAb030038, Atlas Antibodies AB, Stockholm, Sweden) diluted 1:5000. Estrogen receptor (ER) and progesterone receptor (PR) expression were assessed following, heat-mediated antigen retrieval which was performed using microwave treatment for 2 × 5 min in a citrate buffer before being processed in the Ventana Benchmark system (Ventana Medical Systems Inc, AZ) using pre-diluted antibodies to ER (Anti-ER, clone 6F11) and PR (Anti-PgR, clone 16).

### Analysis of immunohistochemical staining

For assessment of nuclear RBM3 expression, both the fraction of positive cells and staining intensity were taken into account using a modification of the previously applied semiquantitative scoring system [16]. Nuclear fraction (NF) was categorized into four groups, namely 0 (0-1%), 1 (2-25%), 2 (26-75) and 3 (> 75%) and nuclear staining intensity (NI) denoted as 0-2, whereby 0 = negative, 1 = intermediate and 2 = moderate-strong intensity. A combined nuclear score (NS) of NFxNI, which had a range of 0 to 6, was then constructed. Cytoplasmic staining intensity was denoted as 0 = negative, 1 = mild and 2 = moderate-strong, and the fraction of positive cells not taken into account. ER and PR negativity was defined as < 10% positively staining nuclei.

### Cell lines and reagents

The human ovarian cancer cell line A2780 and the cisplatin-resistant variant A2780-Cp70 (received as a gift from Prof R Brown, Imperial College, London) were maintained in RPMI-1640 supplemented with glutamine, 10% fetal bovine serum and 1% pencillin/streptomycin in a humidified incubator of 5% CO2 at 37°C. Cisplatin (Sigma-Aldrich, St. Louis, MO, USA) was dissolved in 0.9% NaCl to a stock solution of 1 mg/ml and added to cells to the final concentration (1-100 μM).

### Real-time quantitative PCR and Western Blotting

Total RNA isolation (RNeasy, QIAgen, Hilden, Germany), cDNA synthesis (Reverse Transcriptase kit, Applied Biosystems, Warrington, UK) and quantitative real-time PCR (qRT-PCR) analysis with SYBR Green PCR master mix (Applied Biosystems) were performed as described [25,26]. Quantification of expression levels were calculated by using the comparative Ct method, normalization according to house keeping genes; HMBS (forward primer: 5'-GGC AAT GCG GCT GCA A-3', reverse primer: 5'-GGG TAC CCA CGC GAA TCA C-3'), SDHA (forward primer: 5'-TGG GAA CAA GAG GGC ATC TG-3', reverse primer 5'-CCA CCA CTG CAT CAA ATT CAT G-3') and UBC (forward primer: 5'-ATT TGG GTC GCG GTT CTT G-3', reverse primer: 5'-TGC CTT GAC ATT CTC GAT GGT-3'). For RBM3 amplification, forward primer with sequence 5'-CTT CAG CAG TTT CGG ACC TA-3' and reverse primer with sequence 5'-ACC ATC CAG AGA CTC TCC GT-3' were used. All primers were designed using Primer Express (Applied Biosystems).

For immunoblotting, cells were lysed in ice-cold lysis buffer (150 mM NaCl, 50 mM Tris-HCL pH 7.5, 1% Triton X-100, 50 mM NaF, 1 mM Na3VO4, 1 mM phenylmethylsulfonyl fluoride (PMSF)) and supplemented with protease inhibitor cocktail Complete Mini (Roche, Basel, Switzerland). For Western blotting, 20-50 μg of protein were separated on 15% SDS-PAGE gels and transferred onto nitrocellulose membranes (Hybond ECL, Amersham Pharmacia Biotech, Buckinghamshire, UK). The membranes were probed with primary antibodies followed by horseradish peroxidase (HRP)-conjugated secondary antibodies (Amersham Life Science, Alesbury, U.K.) and visualized using the Enhanced ChemiLuminescence detection system (ECL) and ECL films (Amersham Pharmacia Biotech). RBM3 was detected by the mouse monoclonal anti-RBM3 antibody (AAb030038, Atlas Antibodies AB, Stockholm, Sweden) diluted 1:500 in blocking solution (5% BSA, 1× PBS, 0.1% Tween20), Bax by a polyclonal antibody (BD Pharmingen, San Diego, CA, USA) diluted 1:1000 and Bcl-2 using a monoclonal antibody diluted 1:250 (Santa Cruz, Biotechnology, Santa Cruz, CA, USA). Membranes were stripped and re-probed with an anti-β-actin antibody (Santa Cruz, Biotechnology, Santa Cruz, CA, USA) at a dilution of 1:1000, to provide a loading control.

### Cell pellet arrays

Cell lines were fixed in 4% formalin and processed in gradient alcohols. Cell pellets were cleared in xylene and washed multiple times in molten paraffin. Once processed, cell lines were arrayed in duplicate 1.0 mm cores using a manual tissue arrayer (Beecher Inc, WI) and IHC was performed on 4 μm sections using the RBM3 1B5 antibody diluted 1:1000.

### siRNA knockdown of RBM3 gene expression

Transfection with siRNA against RBM3 (Applied Biosystems, Carlsbad, Ca) or control siRNA (Applied Biosystems) was performed with Lipofectamine 2000 (Invitrogen, Carlsbad, CA) with a final concentration of 50 nM siRNA. All siRNA experiments were performed using three independent RNA oligonucleotides (#58, #59 and #60) targeting RBM3.

### WST-1 cell viability assay

The effect of cisplatin on cell viability was determined by the WST-1 assay (Roche Applied Science, Mannheim, Germany) according to the manufacturer's recommendation. A2780 and A2780-Cp70 cells were seeded in 96-well plates at the density of 2500 cells/well in 100 μl appropriate medium a day before addition of cisplatin. Cells were treated with cisplatin (0-100 μM) for 1 h followed by 24, 48 or 72 hrs recovery in fresh drug-free media. Samples were made in triplicate. Ten microliters WST-1 solution was added per well and incubated at 37°C for 4 hrs. The absorbance of each well was measured using a scanning multiwell spectrophotometer, ELISA reader, at the wavelength of 450 nm and reference wavelength of 690 nm.

### Flow cytometry

For cell cycle phase analysis, cells were fixed in 70% ethanol for 30 minutes at -20°C followed with washing with PBS and centrifugation. In order to label DNA, pellets were resuspended in Vindelöv solution (3.5 μM Tris-HCl pH 7.6, 10 mM NaCl, 10 μg/ml propidium iodide, 20 ug/ml RNase and 0.1% v/v NP40) and incubated in the dark for 20 minutes on ice. The cell cycle analysis was performed by flow cytometry analysis using FACS Calibur (BD Biosciencies, San José, CA), counting in total 1 × 10^4 ^cells. Gating of G0/G1-, S- and G2/M-populations was performed manually using the FlowJo software (version 6.4.7, Tree Star, Inc. Ashland, OR).

The fraction of apoptotic and necrotic cells were analyzed by flow cytometry using AnnexinV-APC and 7AAD (BD Pharmingen, San Diego, CA) staining according to manufacturer's instructions. Briefly, cells were harvested by trypsinization, washed twice in cold PBS and resuspended in 1× Annexin V Binding buffer (BD Pharmingen, San Diego, CA). Cells were stained with AnnexinV-APC antibody and 7AAD and subjected to flow cytometric analysis using a FACSCalibur flow cytometry (BD Biosciencies, San José, CA) to determine the percentage of AnnexinV and 7AAD positive cells. The results are given as the mean of three independent experiments, bars indicate standard error of mean.

### Statistics

Spearman's Rho, Chi-square and Kruskal-Wallis tests were used for comparison of RBM3 expression and relevant clinicopathological characteristics. Kaplan-Meier analysis and log rank test were used to illustrate differences in recurrence free survival (RFS) and overall survival (OS) according to RBM3 expression. Cox regression proportional hazards models were used to estimate the impact of RBM3 expression on RFS and OS in both uni- and multivariate analysis, adjusted for stage and differentiation grade (both cohorts) and volume of residual tumor (0 vs > 0) in Cohort I. Patients who had received neoadjuvant chemotherapy in Cohort I (n = 18) were excluded from the survival analyses. All calculations were performed using SPSS version 15.0 (SPSS Inc, Chicago, IL). All statistical tests were two-sided and a p value < 0.05 was considered statistically significant. The experimental data are expressed as mean ± SEM of at least three independent experiments. Statistical significance of differences between means was determined by one-way ANOVA followed by Duncan's multiple range test or Student's *t*-test.

## Results

### Validation of the RBM3 antibody

**Figure 1:**
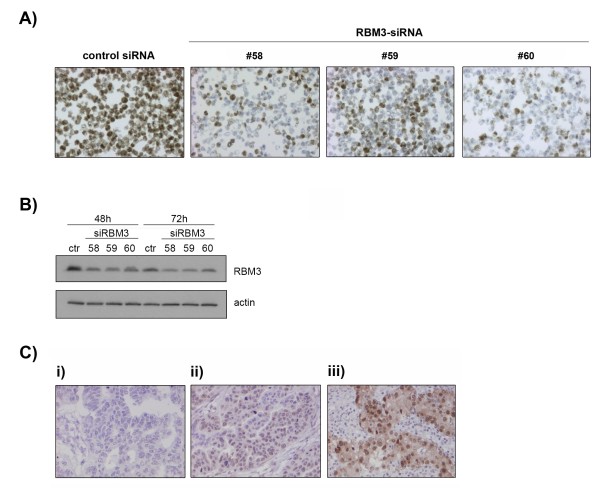
**Specificity of the RBM3 antibody tested in A2780 ovarian cancer cells and immunohistochemical RBM3 expression in primary ovarian tumors**. RBM3 protein expression was significantly decreased after transfection with siRNA against RBM3 in A2780 cells as shown by **(A) **immunocytochemistry 48 hrs post-transfection and **(B) **Western blot 48 and 72 hrs post-transfection. **(C) **Staining of RBM3 was denoted as **(i) **negative (nuclear score = 0), **(ii) **intermediate (nuclear score = 1-2) and **(iii) **strong (nuclear score > 2).

The specificity of the RBM3 antibody was confirmed by siRNA-mediated knockdown of RBM3 in A2780 cells. IHC performed on formalin fixed, paraffin embedded siRNA transfected A2780 cells revealed a marked decrease in immunoreactivity in the RBM3 knockdown cells compared to controls as visualized by IHC on cell pellets (Fig. 1A) and Western blotting (Fig. 1B).

### RBM3 expression and association to clinicopathological characteristics in epithelial ovarian cancer

Having previously demonstrated that RBM3 was associated with a less aggressive breast cancer phenotype [16] we sought to examine the relationship between RBM3 mRNA and protein expression and clinicopathological characteristics in two independent EOC cohorts. In Cohort I, increased RBM3 mRNA levels were not associated with any clinicopathological characteristics (Table 1).

**Table 1:** Correlations between clinicopathological characteristics and RBM3 mRNA (Cohort I) levels and protein expression (Cohort II)

	Cohort I			Cohort II			
RBM3 score	low	high		0	1	2	
n (% for columns)	120 (45.6)	143 (54.4)	p-value	74 (49.0)	50 (33.1)	27 (17.9)	p-value
							
Histological subtype							
mucinuos	0 (0.0)	0 (0.0)	0.887^†^	4 (5.4)	4 (8.0)	3 (11.1)	0.395^†^
serous	111 (92.5)	133 (93.0)		42 (56.8)	31 (62.0)	16 (59.3)	
endometroid	9 (7.5)	9 (6.3)		17 (23.0)	12 (24.0)	5 (18.5)	
clear cell	0 (0.0)	0 (0.0)		6 (8.1)	1 (2.0)	2 (7.4)	
Brenner	0 (0.0)	0 (0.0)		0 (0.0)	0 (0.0)	1 (3.7)	
adenocarcinoma nos	0 (0.0)	1 (0.7)		5 (6.8)	2 (4.0)	0 (0.0)	
							
Differentiation grade							
high	3 (2.5)	14 (9.8)	0.079	2 (2.7)	2 (4.0)	3 (11.1)	0.084
intermediate	40 (33.3)	48 (33.6)		17 (23.0)	13 (26.0)	9 (33.3)	
low	77 (64.2)	78 (54.5)		55 (74.3)	35 (70.0)	15 (55.6)	
missing	0 (0.0)	3 (2.1)		0 (0.0)	0 (0.0)	0 (0.0)	
							
Stage							
I	9 (7.5)	15 (10.5)	0.070	14 (18.9)	7 (14.0)	5 (18.5)	0.760
II	3 (2.5)	15 (10.5)		5 (6.8)	9 (18.0)	3 (11.1)	
III	101 (84.2)	104 (72.7)		40 (54.1)	26 (52.0)	8 (29.6)	
IV	7 (5.8)	9 (6.3)		9 (12.2)	6 (12.0)	7 (25.9)	
missing	0 (0.0)	0 (0.0)		6 (8.1)	2 (4.0)	4 (14.8)	

Correlations were calculated using Spearman correlation test unless other specified.

^†^Kruskal-Wallis test (two-sided).

In Cohort II, following antibody optimisation and staining, it was possible to evaluate the expression of RBM3 protein in 151 cases (98%). Images representing different patterns of expression are shown in Figure 1C. Using the combined score, 57 (38%) tumors lacked RBM3 nuclear RBM3 staining, and 94 (63%) tumors expressed RBM3 in various intensities and fractions. For statistical purposes, tumors were grouped into negative = 0 (combined NS 0-1), intermediate = 1 (combined NS 2-3) and strong = 2 (combined NS > 3). As visualized in Table 1, RBM3 NS was not associated with histological subtype, disease stage or differentiation grade. Cytoplasmic staining was only present in 27 (18%) cases, and therefore not accounted for in the statistics. There was no significant association between RBM3 and ER or PR expression (data not shown).

### Increased RBM3 mRNA levels and protein expression are associated with a prolonged survival in ovarian cancer patients

**Figure 2:**
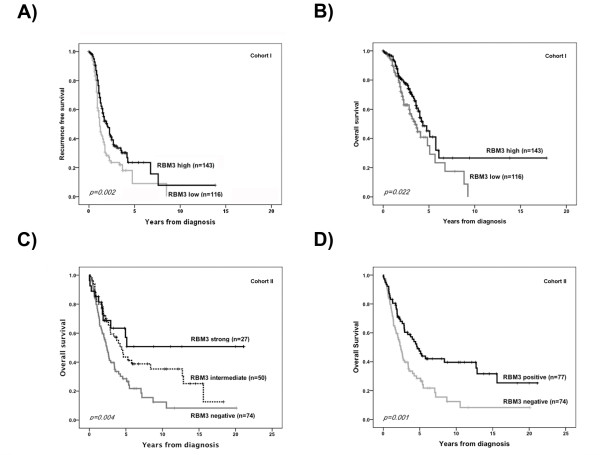
**Increased mRNA (Cohort I) and protein expression (Cohort II) of RBM3 are associated with a prolonged survival**. Kaplan Meier analysis of recurrence free survival **(A) **and overall survival **(B) **according to RBM3 mRNA levels in Cohort I. Kaplan Meier analysis of overall survival according to immunohistochemical RBM3 staining in Cohort II in strata defined as **(C) **negative, intermediate and strong expression and **(D) **negative versus positive expression.

We proceeded to investigate the relationship between RBM3 expression and clinical outcome. In Cohort I, Kaplan Meier analysis demonstrated that increased RBM3 mRNA levels were associated with a significantly prolonged RFS and OS (Fig. 2A and 2B). Cox univariate analysis confirmed this association with an improved RFS (HR = 0.64, 95% CI = 0.47-0.86, *p = 0.003*) and OS (HR = 0.64, 95% CI = 0.44-0.95, *p = 0.024*) (Table 2). Multivariate analysis controlling for age, disease stage, differentiation grade and residual tumor volume (Table 2) confirmed that RBM3 expression was an independent predictor of RFS (HR = 0.61, 95% CI = 0.44-0.84, *p = 0.003*) and OS (HR = 0.62, 95% CI = 0.41-0.95; *p = 0.028*). The independent beneficial prognostic value of RBM3 expression for both RFS and OS was retained when subset analysis of tumours of pure ovarian origin (n = 243) was performed (data not shown).

**Table 2:** Cox uni- and multivariate analysis of recurrence free and overall survival according to RBM3 mRNA levels (Cohort I) and protein expression (Cohort II)

	Recurrence free survival				Overall survival			
		HR (95% CI)		p-value		HR (95% CI)		p-value
								
Cohort I								
			Univariate				Univariate	
low	(n = 115, n_event _= 85)	1.00			(n = 115, n_event _= 54)	1.00		
high	(n = 142, n_event _= 89)	0.64 (0.47-0.86)		0.003	(n = 139, n_event _= 50)	0.64 (0.44-0.95)		0.024
			Multivariate				Multivariate	
low	(n = 103, n_event _= 77)	1.00			(n = 103, n_event _= 51)	1.00		
high	(n = 119, n_even_t = 71)	0.61 (0.44-0.84)		0.003	(n = 117, n_even_t = 50)	0.62 (0.41-0.95)		0.028
								
Cohort II								
							Univariate	
negative	x	x	x	x	(n = 74, n_event _= 33)	1.00		
positive	x	x	x	x	(n = 77, n_event _= 44)	0.53 (0.35-0.79)		0.002
							Multivariate	
negative	x	x	x	x	(n = 68, n_event _= 29)	1.00		
positive	x	x	x	x	(n = 71, n_event _= 42)	0.61 (0.40-0.92)		0.017

Multivariate analysis included adjustment for age (continuous) stage (I-II vs III-IV), grade (I-II vs III) and residual disease (none vs any, only available for Cohort I).

In Cohort II, Kaplan Meier analysis revealed a stepwise association between RBM3 expression and OS whereby RBM3 positive tumors had a significantly improved OS compared to RBM3 negative tumors (Fig. 2C), justifying a dichotomisation into negative versus any expression (Fig. 2D). Cox regression analysis showed a significantly improved OS for RBM3 positive tumors in both univariate (HR = 0.53, 95% CI = 0.35-0.79, *p = 0.002*), and multivariate analysis (HR = 0.61, 95% CI = 0.40-0.92, *p = 0.017*), adjusted for age, stage and differentiation grade (Table 2).

### RBM3 levels are higher in cisplatin-sensitive than cisplatin-resistant ovarian cancer cells

**Figure 3:**
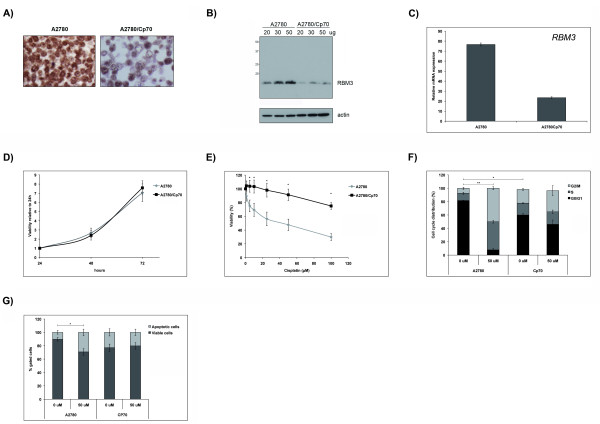
**Expression of RBM3 in the cisplatin-sensitive A2780 ovarian cancer cell line compared to the cisplatin-resistant cell line A2780-Cp70 and the effects of cisplatin treatment on viability, cell cycle phase distribution and apoptosis in A2780 and A2780-Cp70 cell lines**. **(A) **Immunocytochemical staining and **(B) **immunoblotting of RBM3 showing substantially lower RBM3 protein expression in the cisplatin-resistant A2780-Cp70 cell line compared to its parental cisplatin-sensitive A2780 cell line. **(C) **Relative mRNA expression was also reduced in the A2780-Cp70 cells compared to A2780 cells as shown by qRT-PCR analysis. Data shown are mean ± SEM of a representative experiment of two independent experiments performed in triplicate. **(D) **Cell growth illustrated as viability ratio 48:24 and 72:24 hrs measured by WST-1 demonstrating no difference between A2780 and A2780-Cp70 cells. Data shown are mean ± SEM of a representative experiment of three independent experiments performed in triplicate **(E) **Cell viability was evaluated by WST-1 assay in A2780 and A2780-Cp70 cells treated with cisplatin (1, 5, 10, 25, 50 and 100 μM) for 1 h followed by 48 hrs culture in fresh drug-free media. Data are presented as mean values from four independent experiments performed in triplicates presented as percentage of viable cells as compared with untreated cells. Error bars represent SEM. **(F-G) **A2780 and A2780-Cp70 cells were treated with 50 μM cisplatin for 1 h followed by 48 hrs culture in fresh drug-free media followed by flow cytometric analysis of **(F) **cell cycle phase distribution and **(G) **fraction of apoptotic cells. Data are presented as mean value from three independent experiments presented as fold change of cisplatin treatment. Error bars represent SEM.

RBM3 protein expression, assessed by both Western blotting and IHC, was higher in the parental A2780 cells compared to their cisplatin-resistant derivative A2780-Cp70 (Fig. 3A and 3B). Real-time quantitative PCR (qRT-PCR) confirmed a similar difference whereby there was a three fold higher level of RBM3 mRNA in the A2780 compared to the A2780-Cp70 cell line (Fig. 3C). As RBM3 has been found to be associated with proliferation [27,28], we compared the growth rate of the two cell lines, and were unable to demonstrate any significant differences in proliferation (Fig. 3D).

Cell viability assay confirmed cisplatin resistance in the A2780-Cp70 cells relative to the A2780 cells (*p < 0.05*; Fig. 3E). The proportion of cells arrested in G2/M (Fig. 3F) and apoptotic/necrotic cells (Fig. 3G) following cisplatin treatment was also significantly reduced in the A2780-Cp70 cells. Notably, there was a significant difference in cell cycle phase distribution between untreated A2780 and A2780-Cp70 cells (Fig. 3F) with a larger proportion of cells in G2/M in the A2780-Cp70 cells, but no significant difference in apoptosis (Fig. 3G) was observed. However, there was a trend towards a higher fraction of apoptotic cells in untreated A2780-Cp70 cells.

### Downregulation of RBM3 significantly reduces cisplatin sensitivity in ovarian cancer cells

**Figure 4:**
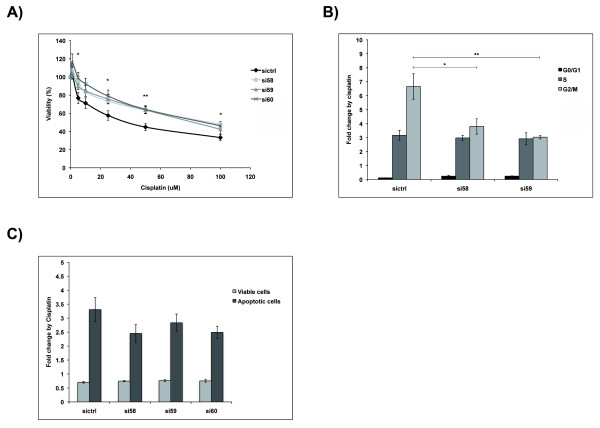
**Down-regulation of RBM3 significantly reduces cisplatin sensitivity in ovarian cancer cells**. siRBM3 transfected A2780 cells were, 24 hrs post-transfection, treated with various concentrations of cisplatin for 1 h followed by 48 hrs culture in fresh drug-free media. **(A) **Cell viability was evaluated by WST-1 assay in siRBM3 transfected A2780 cells treated with cisplatin (1, 5, 10, 25, 50 and 100 μM). Data are presented as mean values from six independent experiments performed in triplicates presented as percentage of viable cells as compared with untreated cells. Error bars represent SEM. **(B) **Cell cycle phase distribution and **(C) **fraction of apoptotic cells were analysed by flow cytometry in siRBM3 transfected A2780 cells treated with 50 μM cisplatin. Data are presented as mean value from four independent experiments presented as fold change of cisplatin treatment. Error bars represent SEM.

The relationship between RBM3 and cisplatin response was then examined using siRNA mediated gene silencing, whereby the cisplatin-sensitive A2780 cells were transfected with three different RBM3-specific siRNA:s or scrambled control siRNA prior to cisplatin treatment. Subsequent determination of cisplatin response as measured by cell cytotoxicity assay and cell cycle analysis revealed that RBM3 knockdown in A2780 cells significantly decreased their sensitivity to cisplatin (Fig. 4A). This effect was evident for three independent RBM3-specific siRNAs. Furthermore, cisplatin induced G2/M arrest was significantly less pronounced in siRBM3 transfected A2780 cells compared to si-control transfected cells (Fig. 4B). There was also a decreased, however non-significant, percentage of apoptotic cells in siRBM3 transfected cells compared to controls (Fig. 4C).

Given the previously demonstrated relationship between RBM3 and apoptosis-regulating proteins [29], we also compared the levels of Bcl-2 and Bax in siRBM3 transfected cells and controls. In line with previous findings [30], Bcl-2 could barely be detected in the A2780 cells while elevated levels were observed in A2780-Cp70 cells. (Additional file 1A). In A2780-Cp70 cells, Bax levels were lower than in A2780 cells, but Bax levels were not considerably altered by down-regulation of RBM3 (Additional file 1B-C).

Cisplatin treatment did not affect RBM3 protein expression or the siRNA mediated down-regulation of RBM3 (Additional file 2). The effects of RBM3 down-regulation in A2780 cells in the absence of cisplatin were also investigated and, in agreement with previous studies [27], siRBM3 transfected A2780 cells showed a significantly reduced cell viability, a slightly higher proportion of cells in G2/M and no effect on apoptosis (Additional file 3).

## Discussion

This investigation of the prognostic value of RBM3 in EOC reveals that RBM3 is an independent prognostic marker at both mRNA and protein levels. Gene expression analysis in a cohort of 267 EOC cases showed that high RBM3 mRNA expression was an independent predictor of a significantly improved RFS and OS. Immunohistochemical analysis in an independent cohort of 154 EOC cases demonstrated that RBM3 protein expression was associated with a significantly improved OS in univariate and multivariate analysis. This is in line with previous findings from two independent breast cancer cohorts where RBM3 was associated with more favourable clinicopathological parameters and a significantly improved survival, irrespective of adjuvant treatment [16]. However, since platinum-based chemotherapy is a fundamental aspect of current EOC treatment regimens, we hypothesized that RBM3 might enhance platinum-sensitivity *in vitro*. We initially confirmed lower RBM3 protein levels in the cisplatin-resistant ovarian cancer cell line A2780-Cp70 compared to their parental cisplatin-sensitive A2780 cells and using RNAi techniques, we demonstrated that silencing of RBM3 led to a decreased cisplatin response in ovarian cancer cells.

Taken together, these data demonstrate that RBM3 is a marker of good prognosis in EOC and a predictor of response to platinum-based chemotherapy, most likely a combination of both, particularly in the light of the previously demonstrated good prognosis associated with RBM3 expression in breast cancer patients, where the vast majority of patients received no adjuvant systemic chemotherapy [16]. While future in-depth studies are warranted to further elucidate the functional mechanisms underlying RBM3's role in cisplatin-mediated cell death, the *in vitro *data presented here provide sufficient evidence to support the hypothesis that, in addition to being a beneficial prognostic biomarker, RBM3 might also predict cisplatin response in EOC. Further studies are required to evaluate the role of RBM3 in predicting response to other platinum based agents, particularly in the setting of a prospective randomised control trial whereby stratification according to different treatment regimens can be performed.

Some aspects on the results presented here merit further attention. The reduced cytotoxic effect of cisplatin in siRBM3 transfected cells was to a large extent reflected by cell cycle alterations, e.g. a lower percentage of cells arrested in G2/M phase rather than by a decreased percentage of apoptotic cells. Cisplatin treatment is known to induce both cell cycle arrest and apoptosis (reviewed in [7]), which was confirmed here in A2780 cells. However, while cisplatin treatment resulted in a significantly decreased cell cycle arrest in siRBM3 transfected A2780 cells, the percentage of apoptotic cells was not significantly reduced. Notably, despite being one of the most cisplatin sensitive ovarian cancer cell lines, A2780 cells have been shown to have a relatively low percentage of apoptotic cells following cisplatin treatment [30]. As there was no evident difference in cell cycle characteristics between siRBM3-transfected and control-transfected A2780 cells without cisplatin treatment, but yet a significantly reduced proportion of siRBM3 treated cells in G2/M-phase arrest following cisplatin treatment, the main effects of RBM3 on cisplatin sensitivity might be reflected in cell cycle distribution rather than apoptosis. Although RBM3 mRNA expression has previously been associated with the pro-apoptotic Bax gene in breast cancer [29], we were unable to demonstrate a down-regulation of Bax protein in siRBM3 transfected A2780 cells, further supporting the theory that RBM3 promotes cisplatin sensitivity through cell-cycle regulation.

It is evident that RBM3 is up-regulated in response to various conditions causing cellular stress, i.e. hypothermia [13,31,32] hypoxia [26], serum starvation [28] and exposure to microgravity [33]. We did not see an up-regulation of RBM3 upon cisplatin-induced stress, however, it would still be of interest to investigate the potential role of RBM3 in DNA repair response given the observed decrease of RBM3-silenced cells arrested in G2/M upon cisplatin treatment.

Apart from our previous study in breast cancer [16], there are to our knowledge no other published data on the prognostic impact of tumor-specific RBM3 expression in human cancer. However, RBM3 has been identified as one of five down-regulated genes in an *in vitro *model of melanoma progression [34], implying a beneficial impact on prognosis also in this cancer form. Contrasting *in vitro *data have also been published, whereby RBM3 has been proposed to be a proto-oncogene stabilizing COX-2 mRNA levels and protecting against mitotic catastrophe in colorectal cancer cell lines [27].

A common observation in human tissue is that RBM3 is up-regulated in cancer [27,28] and in proliferating non-malignant cells [28], compared to normal cells. Quite in line with these findings, we found that siRNA mediated knockdown of RBM3 resulted in reduced proliferation in A2780 cells, which might in part explain the reduced cisplatin sensitivity and also the relatively modest reduction in apoptosis upon treatment in siRBM3 transfected A2780 cells. Yet, as a consequence of the observation that RBM3 seems to be necessary for the maintenance of cellular integrity during various stress conditions, it has been hypothesized that targeting RBM3 could prove to be an efficient novel therapeutic strategy against cancer [27,35]. This viewpoint is challenged by the results presented here, but, evidently, drug induced effects by RBM3 modulation seem to differ between different cell line models, as down-regulation of RBM3 has been associated with enhanced response to adriamycin and cisplatin in androgen dependent but not androgen-independent prostate cancer cells [35]. To what extent this variation is true in human tumors remains to be elucidated but to our knowledge, in contrast to ovarian cancer, prostate cancer is not routinely treated with platinum-based chemotherapeutic agents. It should also be emphasized that, in a translational context, the proposal that RBM3 is a proto-oncogene activated in response to adverse cellular conditions [27] does not contradict the findings that its presence in an established tumor is associated with a favourable patient outcome as such findings would not have taken patient treatment into account.

The coincidence of a beneficial prognostic impact of RBM3 expression in EOC both at the mRNA and protein level demonstrated here is particularly relevant from a translational perspective as it would justify using IHC, which is a simpler, faster and less costly method than RT-PCR in the clinical setting. In our previous study in breast cancer [16], the favourable prognostic impact of RBM3 was assessed by IHC in two independent patients cohorts using a polyclonal, monospecific antibody, initially developed within the HPA programme [23,36]. In the present study, the favourable prognostic impact of RBM3 expression in EOC was demonstrated at both the gene expression and protein levels in two relatively large independent cohorts. RBM3 protein expression was assessed using a monoclonal antibody which displayed a single band of the expected size on Western blot and further validation showed a decreased RBM3 expression in siRNA transfected A2780 cells compared to controls, both as assessed by IHC and Western blotting. Notably, the previously used polyclonal antibody has also been validated in the A2780 cells with similar results to the monoclonal antibody; e.g. differential expression in A2780 and A2780-Cp70 cells and decreased expression in siRNA transfected cells (data not shown). Furthermore, analysis of the tumor specimens in Cohort II using the antibody that was used in the breast cancer study [16] yielded concordant results regarding the prognostic impact of tumor-specific RBM3 expression (data not shown).

In breast cancer, nuclear RBM3 expression was associated with favourable clinicopathological parameters, including hormone receptor status [16]. In this study, we found no association between RBM3 and ER or PR expression in EOC as assessed by IHC. This observation indicates that RBM3 might have different functions in the context of estrogen-related signalling in breast cancer and ovarian cancer. The potential clinical relevance of this is however less evident as the beneficial effect of high RBM3 expression in breast cancer was independent of tamoxifen treatment.

## Conclusions

Here, we present data from two independent patient cohorts demonstrating that expression of the RNA-binding protein RBM3, both at the mRNA and protein levels, is associated with a good prognosis in epithelial ovarian cancer. Furthermore, we show that decreased RBM3 expression confers reduced platinum sensitivity in ovarian cancer cells. These findings indicate that RBM3 may be a useful prognostic and treatment predictive marker in epithelial ovarian cancer.

## Competing interests

The authors declare that they have no competing interests.

## Authors' contributions

ÅE participated in the data collection, performed the statistical analysis, carried out the functional studies and drafted the manuscript. DB participated in the data collection, performed statistical analysis, and helped to draft the manuscript. BN constructed the TMAs and participated in the data collection. DPO participated in the data collection. JE participated in the design of the study and helped draft the manuscript. MAK assisted with the data collection and helped draft the manuscript. IBJ assisted with the statistical analysis. JM assisted with data collection and helped to draft the manuscript. JB assisted with collection of clinical data. MU assisted with data collection and participated in its design. FP assisted with data collection and helped to draft the manuscript. KJ conceived of the study, participated in its design and coordination and helped to draft the manuscript. All authors read and approved the final manuscript.

## Supplementary Material

Additional file 1 **Expression of the apoptosis regulating proteins Bcl-2 and Bax in A2780 and A2780-Cp70 cells and siRBM3 transfected A2780 cells compared to controls**. Western blot analysis of **(A) **Bcl2 expression in A2780, A2780-Cp70 and siRBM3 transfected A2780 cells and Bax expression in **(B) **A2780 and A2780-Cp70 cells and **(C) **siRBM3 transfected A2780 cells. Click here for file

Additional file 2 **Cisplatin treatment does not affect the protein level of RBM3 or the siRNA-mediated down-regulation of RBM3**. **(A) **Protein expression of RBM3 was examined by immunoblotting in A2780 cells treated with various concentrations of cisplatin for 1 h followed by 48 hrs culture in fresh drug-free media. **(B) **siRBM3 transfected A2780 cells were, 24 hrs post-transfection, treated with 50 μM cisplatin for 1 h followed by 48 hrs culture in fresh drug-free media whereby RBM3 remained down-regulated as shown by immunoblotting. Click here for file

Additional file 3 **The effects of RBM3 down-regulation on cell viability, cell cycle characteristics and apoptosis in A2780 cells**. **(A) **Cell viability was evaluated by WST-1 assay in siRBM3 transfected A2780 cells. Data are presented as mean values from five independent experiments performed in triplicates presented as percentage of viable cells relative to si-control transfected cells. Error bars represent SEM. **(B) **Cell cycle phase distribution and **(C) **fraction of apoptotic cells were analysed by flow cytometry in siRBM3 transfected A2780 cells. Data are presented as mean value from four independent experiments. Error bars represent SEM. Click here for file
