# Supplementary figures and images for: Expression of the RNA-binding protein RBM3 is associated with a favourable prognosis and cisplatin sensitivity in epithelial ovarian cancer
Source: J Transl Med. 2010 Aug 20;8:78. doi: 10.1186/1479-5876-8-78 (PMC2936876; doi:10.1186/1479-5876-8-78)

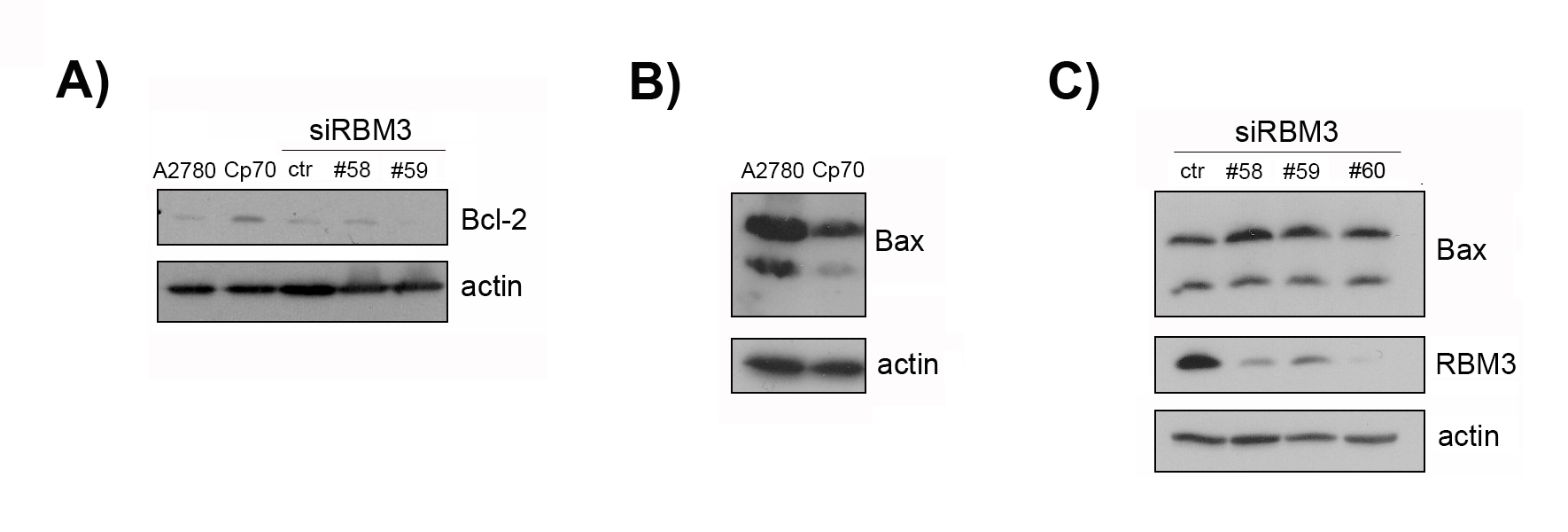

Supplement: Additional file 1 — Expression of the apoptosis regulating proteins Bcl-2 and Bax in A2780 and A2780-Cp70 cells and siRBM3 transfected A2780 cells compared to controls. Western blot analysis of (A) Bcl2 expression in A2780, A2780-Cp70 and siRBM3 transfected A2780 cells and Bax expression in (B) A2780 and A2780-Cp70 cells and (C) siRBM3 transfected A2780 cells. [file 1479-5876-8-78-S1.JPEG]

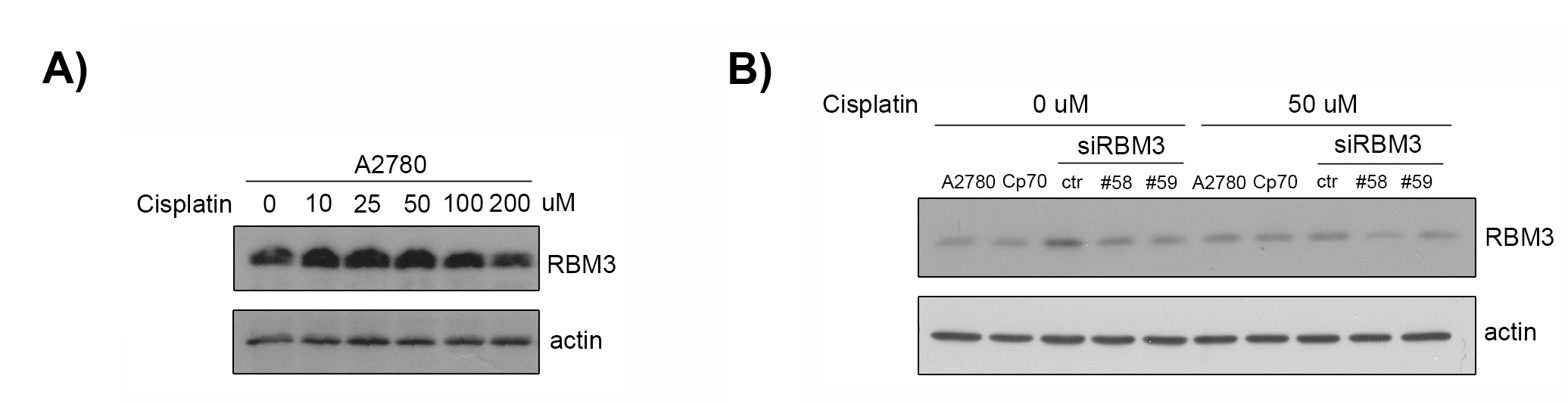

Supplement: Additional file 2 — Cisplatin treatment does not affect the protein level of RBM3 or the siRNA-mediated down-regulation of RBM3. (A) Protein expression of RBM3 was examined by immunoblotting in A2780 cells treated with various concentrations of cisplatin for 1 h followed by 48 hrs culture in fresh drug-free media. (B) siRBM3 transfected A2780 cells were, 24 hrs post-transfection, treated with 50 μM cisplatin for 1 h followed by 48 hrs culture in fresh drug-free media whereby RBM3 remained down-regulated as shown by immunoblotting. [file 1479-5876-8-78-S2.JPEG]

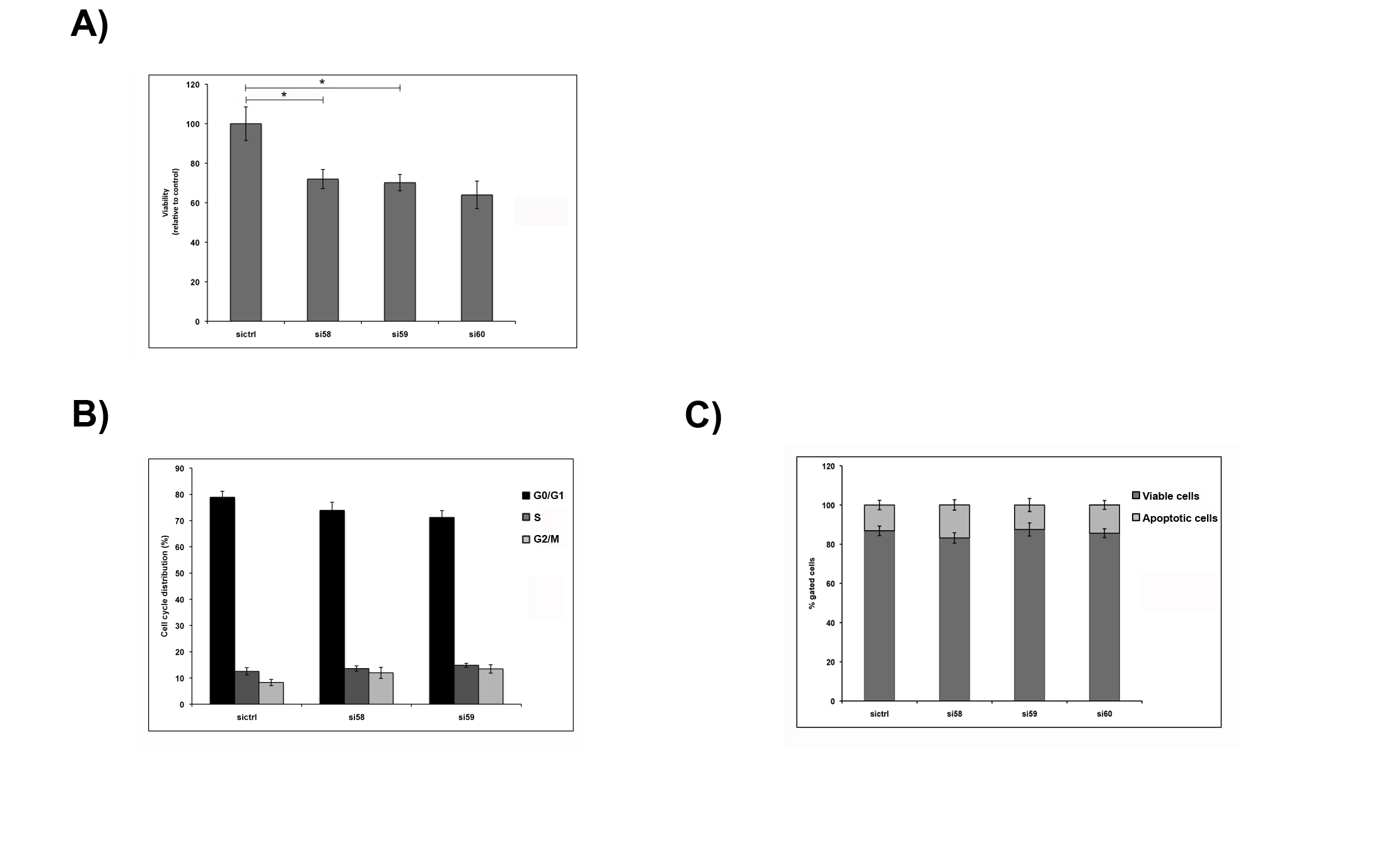

Supplement: Additional file 3 — The effects of RBM3 down-regulation on cell viability, cell cycle characteristics and apoptosis in A2780 cells. (A) Cell viability was evaluated by WST-1 assay in siRBM3 transfected A2780 cells. Data are presented as mean values from five independent experiments performed in triplicates presented as percentage of viable cells relative to si-control transfected cells. Error bars represent SEM. (B) Cell cycle phase distribution and (C) fraction of apoptotic cells were analysed by flow cytometry in siRBM3 transfected A2780 cells. Data are presented as mean value from four independent experiments. Error bars represent SEM. [file 1479-5876-8-78-S3.JPEG]
